# Comment on: Utility of QuantiFERON tuberculosis gold test in a south Indian patient population of ocular inflammation

**DOI:** 10.4103/0301-4738.64121

**Published:** 2010

**Authors:** Jay Chhablani

**Affiliations:** Smt. Kanuri Santhamma Retina Vitreous Centre, L V Prasad Eye Institute, Kallam Anji Reddy Campus, L V Prasad Marg, Banjara Hills, Hyderabad - 500 034, Andhra Pradesh, India

Dear Editor,

We have read with interest the article by Babu *et al*., "Utility of QuantiFERON TB gold test in a south Indian patient population of ocular inflammation".[1]

It is a good attempt made in Indian patients to find out the utility of the QuantiFERON TB gold test (QFT). I wish to present certain doubts and make a few comments.

1. For Group A, patients were considered true negative (disease-free) only on the basis of no history of exposure to systemic tuberculosis (TB) or history of active TB. They could be asymptomatic or subclinical TB patients. In patients who were positive (number =5/21) with QFT, systemic evaluation should have been done before considering them false positive.
2. Author has not included history of *Bacille Calmette-Guerin* (BCG) vaccination which can cause false positivity of the positive purified protein derivative (PPD) test.[2]
3. The statement "Comparing Groups C and D, the sensitivity of the QuantiFERON TB gold test to pick up intraocular TB was 82% while the specificity of the QuantiFERON TB gold test in intraocular TB was 76%", Group C is considered as without intraocular TB only on the basis of clinical findings and no history of exposure to active TB. Although two patients were positive to both PPD and QFT, they were considered as false positive, which needs further evaluation.
4. The statement "The number of cases in Groups C and D who were both PPD test and QuantiFERON TB gold tests positive and who had intraocular TB was 29", if the author has considered Group D as presumed intraocular TB , then this number should be at least 36.
5. The statement "the number of cases who were both PPD test and QuantiFERON TB gold tests positive and who did not have intraocular TB was one", if the author has considered that Group C is without intraocular TB then this number should be two.

On the basis of the above two statements the sensitivity and specificity of the combination of PPD and QFT in the diagnosis of intraocular TB changes. This needs some clarification.
